# Social Housing Conditions Modulate the Long-Lasting Increase in Cocaine Reward Induced by Intermittent Social Defeat

**DOI:** 10.3389/fnbeh.2019.00148

**Published:** 2019-07-04

**Authors:** Carmen Ferrer-Pérez, Marina D. Reguilón, Carmen Manzanedo, José Miñarro, Marta Rodríguez-Arias

**Affiliations:** Department of Psychobiology, Faculty of Psychology, Universitat de València, Valencia, Spain

**Keywords:** social defeat, oxytocin, cocaine, conditioned place preference, IL-6, social environment

## Abstract

Social defeat is considered the most representative animal model for studying the consequences of social stress. Intermittent social defeat (ISD) has proved to enhance the response to cocaine hedonic properties. In the present research, we evaluated if different social housing conditions, as housing with a familiar conspecific or with a female, exert a protective effect modulating the negative consequences of ISD as the increased sensitivity to cocaine and the induction of anxiety-like behavior. To achieve this objective, non-stressed or ISD OF1 male mice were divided into five different experimental groups according to their social environment: standard housing (four adult males per cage); male adolescent or adult in pairs (two males per cage); and adult males housed with a female for a short or long period (3 days vs. the whole duration of the study). Anxiety-like behavior was evaluated 19 days after the last episode of ISD using an elevated plus maze (EPM), and 24 h later the animals underwent a conditioned place preference paradigm (CPP) induced by a sub-threshold dose of cocaine (1 mg/kg). Following CPP, biological samples were taken to measure striatal levels of interleukin 6 (IL-6) and plasmatic levels of oxytocin (OT). Our results confirmed that ISD animals housed in standard condition displayed an anxious phenotype, developed CPP and had increased levels of IL-6 in the striatum. However, animals housed with a female or with a familiar male since adolescence did not develop CPP and were protected against the anxiogenic and neuroinflammatory potential of ISD stress. In the group of animals paired with a female throughout the experimental procedure, an increase in OT levels may have underlain this buffering effect, while the protective effect of being housed with a familiar male mouse seems to be related with a better resolution of the stress response. The present results expand our knowledge of the neurobiology of vulnerability to drug addiction and highlight the benefit of social support for recovery from the adverse effects of social stress.

## Introduction

Drug addiction is a chronic disorder characterized by loss of control over the use of a substance and relapse during cessation attempts (Koob and Volkow, 2010; Volkow and Morales, 2015). The development of substance use disorder (SUD) is multifactorial, and the vulnerability to develop an addiction depends on a complex interplay between biological and environmental factors (Strickland and Smith, 2014).

Among environmental influences, social factors are powerful determinants of behavior and health status (Kessler et al., 2010; Ajonijebu et al., 2017). In this regard, there is a growing interest among researchers in studying the influence of social factors in addictive disorders (Neisewander et al., 2012). Although social stimuli can act as positive natural reinforcers that compete with drug reward, other social interactions can be highly challenging and become stressors (Heilig et al., 2016). For instance, social experiences with a negative affective valence (isolation or bulling in the workplace) are linked with higher rates of drug abuse and vulnerability to relapse after periods of detoxification (Sullivan et al., 2006; Niedhammer et al., 2010). On the other hand, positive social environments, such as strong family ties, involvement and attachment, are associated with lower rates of drug use and better prognosis during treatment (Stout et al., 2012; Litt et al., 2016).

Basic research with animal models using social and hierarchic status has highlighted the dual role of social factors in addiction. Animals living in social environments that provide access to socially rewarding experiences, such as sexual behaviors and pair bonding, are protected against drug-related behaviors (Beloate and Coolen, 2017; Rodríguez-Ortega and Cubero, 2018). For instance, a study carried out with socially housed rodents that acquired cocaine self-administration (SA) behavior and then experienced a forced period of abstinence showed a lower risk of displaying cue-elicited cocaine-seeking behavior than socially isolated animals that underwent the same experimental procedure (Thiel et al., 2010). Similarly, group-housed animals showed a lower risk of drug- or stress-induced reinstatement of cocaine conditioned place preference (CPP) in a former research carried out in our laboratory (Ribeiro Do Couto et al., 2009). On the other hand, social stressor experiences have repeatedly been reported to enhance the response to drugs, to escalate drug consumption and to promote relapse (see revision in Neisewander et al., 2012; Montagud-Romero et al., 2016). For example, early-life social stress experiences, like poor maternal care or maternal separation, have shown to increase ethanol and cocaine consumption in rats in different studies (Francis and Kuhar, 2008; Isengulova et al., 2009). Among all the paradigms that model social stress in rodents, such as social deprivation, social instability, and territorial and maternal aggression, social defeat is considered the most representative for studying the physiological and behavioral consequences (Neisewander et al., 2012; Hammels et al., 2015). This paradigm closely mimics the reality of subordinate vs. aggressor relations in humans (Björkqvist, 2001; Selten et al., 2013), and its ecological validity is widely demonstrated (Miczek et al., 2008). Also named the resident-intruder paradigm, it is based on the territorial attack of a resident male confronted with a conspecific intruder. In these agonistic encounters, residents and intruders demonstrate natural offensive and defensive behaviors, which allows researchers to study the short- and long-term behavioral and physiological consequences of social defeat stress. Overall, the scientific literature affirms that experiences of repeated or intermittent social defeat (ISD) enhance the unconditioned and conditioned rewarding responses to psychostimulant drugs and precipitate the reinstatement of drug seeking in the SA and CPP paradigms, while chronic social defeat produces the opposite effects, with animals displaying a decreased tendency to consume cocaine (see revision in Neisewander et al., 2012; Shimamoto, 2018).

Several neurobiological theories have been proposed to explain stress-induced vulnerability, including alterations of corticotrophin-releasing factor (CRF; Ferrer-Pérez et al., 2018b), dopamine neurotransmission system (Reguilón et al., 2017) and epigenetic forms of plasticity (Montagud-Romero et al., 2016; Ajonijebu et al., 2017). Recent studies suggest that inflammatory processes mediate the effect of ISD stress with regard to an enhanced drug response and anxiety-like behavior (Ferrer-Pérez et al., 2018a). Chronic social defeat and ISD promote the activation of the immune system and trigger a pro-inflammatory state characterized by increased levels of cytokines such as interleukin IL-1β or IL-6 (Wohleb et al., 2011, 2013, 2014; Hodes et al., 2014; Stankiewicz et al., 2015; Pfau and Russo, 2016; Ferrer-Pérez et al., 2018a), which has also been reported to compromise the integrity of the brain blood barrier (Rodríguez-Arias et al., 2017). Within the framework of this theory of neuroinflammation, some researchers have explored anti-inflammatory interventions as therapeutic targets in stress-related disorders, which have proven to be effective in reversing cognitive impairments, anxiety-like behavior and the enhancement in cocaine response induced by social stress (Pfau and Russo, 2016; Duque et al., 2017; Ferrer-Pérez et al., 2018a).

Positive social environments have been reported to have a protective effect on SUD development. Oxytocin (OT), is a neuropeptide that is released during physical contact and potentiates social behaviors (Carter, 2003). It might be central explaining the buffering effect of positive social environments as it has a direct effect reducing the activity of the hypothalamic-adrenal-axis (HPA) during stress response (Lee et al., 2009). Additionally, several studies have revealed anti-inflammatory and antioxidant properties of OT (Karelina et al., 2011; Yuan et al., 2016). In fact, it has shown to be effective in attenuating behavioral and physiological consequences of social stressors such as isolation, and has proven to be effective in reversing depressive and anxiety-like behaviors (Windle et al., 1997; Grippo et al., 2012). In the present research, we have analyzed if the long-lasting negative consequences of ISD on the anxiety-like phenotype and cocaine response can be reversed by different positive social housing conditions (e.g., pairing with a familiar conspecific or a female). Additionally, we aimed to determine if the physiological mechanism that underlies this protective effect is linked to an anti-inflammatory effect of social intervention that could be mediated by the release of OT. Increasing our knowledge of how social context contributes to responses to drugs can lead to new avenues of drug prevention and treatment.

## Materials and Methods

### Animals

A total number of 195 OF1 mice were supplied by Charles Rivers (France). The mice were divided into groups of 92 adults (42 days old) and 24 adolescents (21 days old). On arrival at the animal facility, the experimental mice were housed in groups of four in plastic cages (27 × 27 × 14 cm), with the exception of 48 animals (24 adults and 24 adolescents) that were housed in pairs. In addition to the experimental mice, 44 adult OF1 females were employed to provide female-paired housing. Finally, 35 adult male OF1 mice, to be used later as residents in the social defeat encounters, were housed individually in plastic cages (21 × 32 × 20 cm) for a month prior to the experiments in order to induce heightened aggression (Rodríguez-Arias et al., 1998).

Regardless of the experimental group to which they were assigned, all the animals were kept under the same conditions: constant temperature; a reversed light schedule (white light on 8:00–20:00 h); and food and water available ad libitum, except during behavioral tests. The experimental protocol was approved by an Institutional Review Committee for the use of animal subjects (Comité d’Ética d’Experimentació i Benestar Animal, number 2015/VSC/PEA/00168). Procedures involving mice and their care were conducted according to national, regional and local laws and regulations, which are in compliance with the Directive 2010/63/EU. Every effort was made to minimize the animals’ suffering and reduce the number of animals used.

### Drugs

For CPP conditioning, animals were injected intraperitoneally with a dose of 1 mg/kg of cocaine hydrochloride (Alcaliber Laboratory, Spain) dissolved in physiological saline (NaCl 0.9%) and adjusted to a volume of 0.01 ml/g of weight. This dose of cocaine was selected on the basis of previous CPP studies (Montagud-Romero et al., 2016; Ferrer-Pérez et al., 2018a,b) showing 1 mg/kg to be a sub-threshold dose for inducing CPP in adult animals without previous stress or drug experiences and housed in standard condition.

### Experimental Groups and Experimental Design

**Figure 1:**
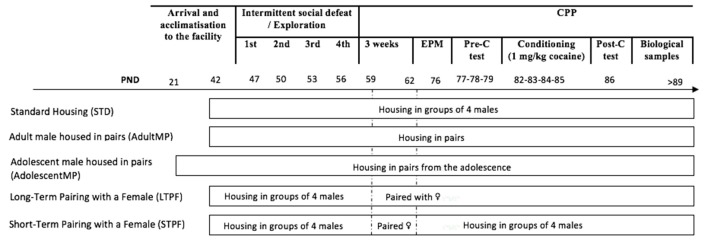
Experimental design. The experimental mice were divided into five different groups according to housing conditions. Animals in the Standard Housing group were housed in groups of four male mice per cage during the whole experiment (STD-nonISD *n* = 12 and STD-ISD *n* = 12). A second group of animals (AdultMP) were housed in pairs upon arrival at the animal facility on PND 42 (AdultMP-nonISD *n* = 12 and AdultMP-ISD *n* = 12). Similarly, a third group of adolescent mice (AdolescentMP) was housed in pairs on their arrival at the laboratory on PND 21 (AdolescentMP-nonISD *n* = 12 and AdolescentMP-ISD *n* = 12). Animals in the Long-Term Pairing with a Female (LTPF) housing condition were initially housed in the same way as those in the STD group, until the end of ISD protocol. Subsequently, 48 h after the last episode of social defeat, they were rehoused in a new cage with a female until the end of the experiments (LTPF-nonISD *n* = 12 and LTPF-ISD *n* = 12). Likewise, animals in the Short-Term Pairing with a Female (STPF) housing condition underwent the same housing procedure as LTPF animals, only that they were housed with the female for 72 h, after which, the experimental animals were returned to the original cage and regrouped as animals in standard housing groups (STPF-nonISD *n* = 8 and STPF-ISD *n* = 12).

Mice were divided into different experimental groups (depicted in Figure 1) based on housing conditions. Next, half of the animals in each housing condition underwent an ISD, while the other half underwent a similar manipulation procedure without the experience of social defeat (nonISD). Subsequently, 19 days after the last social defeat, anxiety was evaluated in the elevated plus maze (EPM) test. One day later, the CPP procedure was initiated. Biological samples were taken after the CPP protocol on PND >89.

### Apparatus and Procedures

#### Intermittent Social Defeat (ISD) Procedure

The ISD protocol followed in this study has been widely validated as a social stressor (Hodes et al., 2014; Hammels et al., 2015) and has been described in detail in previously published research by our group (Ferrer-Pérez et al., 2018a,b). Five days prior to initiation of the ISD protocol, aggressive residents were screened to confirm appropriate levels of aggressive behavior. The aggression test was performed in the home cage of the resident by placing an intruder adult OF1 mice in the cage for 3 min. Any resident mouse showing a latency to attack of over 3 min was withdrawn from the experiment.

The social defeat episodes consisted of three phases, each of which began by introducing the “intruder” (the experimental animal) into the home cage of the “resident” (the aggressive opponent) for 10 min. During this initial phase, the intruder was protected from attack, but the wire mesh walls of the cage allowed for social interaction and species-typical threats from the aggressive male resident, thus leading to instigation and provocation. The wire mesh was then removed from the cage to allow physical contact between the two animals for a 5-min period. In the third phase, the wire mesh was put in place again to separate the two animals for another 10 min while allowing social threats by the resident. Intruder mice were exposed to a different aggressor during each episode of social defeat. The criterion used to define an animal as defeated was the adoption of a specific posture signifying defeat, characterized by an upright submissive position, limp forepaws, upwardly angled head, and retracted ears. In order to minimize physical wounding during social defeats, the 5-min direct encounters were interrupted if the intruder displayed a submissive supine posture for more than 8 s or if it was bitten by the aggressor more than 12 times. All agonistic encounters were videotaped to confirm social defeat. The nonISD groups followed the same protocol, but without the presence of a “resident” mouse: the mouse was placed in a new cage enclosed with a wire mesh for 10 min, after which the mesh was removed for 5 min and then returned for the last 10 min of each exploration session.

#### Conditioned Place Preference (CPP)

The CPP protocol consisted of three phases and took place during the dark cycle following an unbiased procedure in terms of initial spontaneous preference. For place conditioning, we employed sixteen identical Plexiglas boxes with black and white equal sized compartments (30.7 × 31.5 × 34.5 cm) separated by a gray central area (13.8 × 31.5 × 34.5 cm). In brief, during preconditioning (Pre-C), the time spent by the animal in each compartment over a 15-min period was recorded. Mice showing a strong unconditioned aversion (less than 33% of the time spent in both compartments) or preference (more than 67%) for any compartment were excluded from the study.

In the second phase (conditioning), animals underwent two pairings per day. First, they received an injection of physiological saline before being confined to the vehicle-paired compartment for 30 min. After a 4-h interval, they received cocaine immediately before being confined to the drug-paired compartment for 30 min. In the third phase (post-conditioning; Post-C) the conditioned preference was assessed by measuring the time spent by mice in a drug-free state in each compartment during the 15-min observation period. The difference in seconds between the time spent in the drug-paired compartment in the Post-C test and that spent in the Pre-C test is an estimation of the degree of conditioning induced by the drug. If this difference is positive, then the drug is considered to have induced a preference for the drug-paired compartment, whereas the opposite indicates the development of aversion. Additionally, a conditioning score (CS) was calculated for each mouse based on the difference between the time spent in the drug-paired compartment during the Post-C and Pre-C tests. If this difference is positive, then the drug is considered to have induced a preference for the drug-paired compartment, whereas the opposite indicates the induction of an aversion.

#### Elevated Plus Maze-EPM

The EPM test was carried out essentially following the procedure described by Daza-Losada et al. (2009). The maze consisted of two open arms (30 × 5 × 0.25 cm) and two enclosed arms (30 × 5 × 15 cm), and a central platform (5 × 5 cm) elevated 45 cm above floor level. In order to decrease experimental stress, animals were habituated to the experimental room for 1 h prior to testing. At the beginning of each trial, experimental mice were placed on the central platform so that they were facing an open arm and were allowed to explore for 5 min. The behavior displayed by the mice during the test was recorded by an automated tracking system (EthoVision 3.1, Noldus) that tracks the number of entries and time spent in each section of the maze (open arms, closed arms, central platform). The time and percentage of time spent in the open arms were measured to characterize the anxiolytic effects of the different social housing conditions (Bourin et al., 2007; Blanco-Gandía et al., 2018).

#### Tissue Sampling

Animals were sacrificed by cervical dislocation and then decapitated to collect blood from the neck in tubes coated with heparin. Blood samples were kept on ice, and plasma was separated from whole blood by centrifugation (5 min, 5,000 G) and transferred to sterile 0.2 ml microcentrifuge tubes. To obtain striatum samples brains were removed immediately after decapitation and dissected following the procedure described by Heffner et al. (1980). Plasma and tissue samples were stored at −80°C until IL-6 and OT determinations.

#### Determination of Striatal IL-6 and Plasmatic Oxytocin Levels

To determine striatal IL-6 concentration we used a Mouse IL-6 ELISA Kit obtained from Abcam (Ref: Ab100712) following the manufacturer’s instructions. Before running the kit, striatum samples were first homogenized and prepared following the procedure described in detail by Ferrer-Pérez et al. (2018a), and protein levels were determined by the Bradford assay from ThermoFisher (Ref: 23227).

For the quantification of plasmatic OT, we used an ELISA kit from Arbor Assays (Ref: K048-H1). Following the recommendation of Leng and Sabatier (2016), we performed an extraction procedure to reduce the non-specific binding of plasmatic proteins in our samples. The extraction procedure was carried out using the extraction solution and the protocol provided by the ELISA kit manufacturer (Arbor Assays). ELISA test results were read using an iMark microplate reader (Bio-RAD) controlled by Microplate Manager 6.2 software, and the final results were expressed in pg/mg for striatal tissue samples and in pg/ml for plasma.

### Statistical Analyses

A preliminary three-way analysis of variance (ANOVA) was carried out with the CPP data of animals under positive social housing condition with two between-subjects variables—Stress, with two levels (ISD and nonISD), and Housing, with four levels (AdultMP, AdolescentMP, LTPF, STPF)—and a within-subjects variable—Days, with two levels (Pre-C and Post-C). The preliminary statistical analysis carried out with the CPP data showed that animals housed in pairs with a female for a short (STPF) or a long term (LTPF) had equivalent results in this test. As a consequence, they were pooled in one single group (pairing with a female, PF) in further analyses. Additionally, the group of adult animals housed in pairs with other males (AdultMP) were removed from further experiment analyses as this intervention showed non-protective effects and both EXP and ISD animals displayed CPP. Outcomes of this group in each test are available as Supplementary Material (see Supplementary Table S1). Taking these results into account, subsequent ANOVAs included only three intervention groups: standard housed, adolescent male paired, and paired female.

A two-way ANOVA with the aforementioned between-subjects variables (Stress and Housing) was employed to analyze the data of EPM, IL-6, and OT levels. The value of the effect size was evaluated using partial eta-squared. Data are presented as mean ± standard error of the mean (SEM) and a *p*-value < 0.05 was considered statistically significant. Analyses were performed using SPSS v24. In all cases, *post hoc* comparisons were performed with Bonferroni tests.

## Results

### Housing Conditions Decrease ISD-Induced Anxiogenic Behavior Evaluated in the EPM

**Figure 2:**
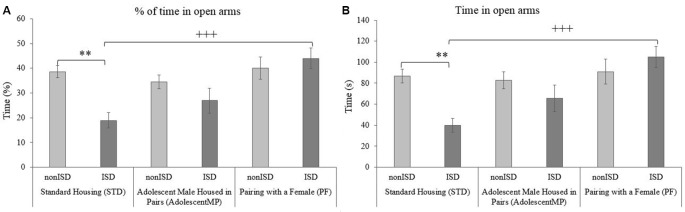
Positive housing conditions decrease ISD-induced anxiogenic behavior evaluated in the elevated plus maze (EPM). **(A)** Percentage of time spent in the open arms of the EPM. **(B)** Time (s) spent in the open arms of the EPM. **p < 0.01 significant difference between STD-nonISD vs. STD-ISD. ^+++^*p* < 0.001 significant difference between STD-ISD vs. PF-ISD. Data presented as mean values ± standard error of the mean (SEM).

The ANOVA of the EPM data (see Figure 2) revealed an effect of the interaction Housing × Stress on the time spent in the open arms (*F*_(2,86)_ = 4.454; *p* = 0.014; effect size 0.094) and on the percentage of open *entries* (*F*_(2,86)_ = 4.304; *p* = 0.017; effect size 0.091). ISD mice housed under the standard condition spent less time and less percentage of time in the open arms than their corresponding non-stress controls (nonISD; *p* < 0.01) and compared to animals housed with a female for long and short terms (PF; *p* < 0.001).

### Housing Conditions Modulate the Increase in the Cocaine-Conditioned Reward Induced by ISD Stress

**Figure 3:**
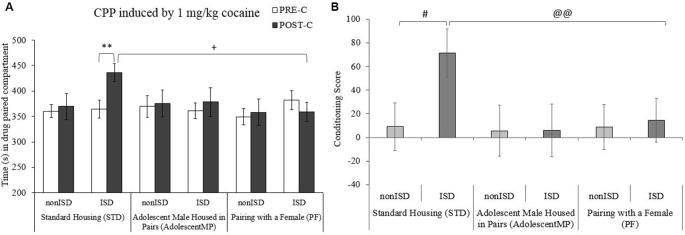
Positive housing conditions decrease the effects of ISD stress on the acquisition of 1 mg/kg cocaine-induced conditioned place preference (CPP). Bars represent the following groups: standard housing (STD-nonISD *n* = 12; STD-ISD *n* = 12); male animals housed in pairs since adolescence (AdolescentMP-nonISD *n* = 12; AdolescentMP-ISD *n* = 12); male animals paired with a female for long or short term (PF-nonISD = 20, PF-ISD = 20). **(A)** The bars represent the time (s) spent in the drug-paired compartment in the PRE-C test (before conditioning sessions; white bars), and in the POST-C test (after conditioning sessions; black bars). Data are presented as mean values ± SEM. Bonferroni’s test **p < 0.01 significant difference in the time spent in the drug-paired compartment in POST-C vs. PRE-C in STD-ISD animals. ^+^*p* < 0.05 significant differences in the POST-C time between STD-ISD vs. PF-ISD. **(B)** Bars represent the conditioning score (CS), calculated as the time spent in the drug-paired compartment in the Pre-C test minus the time spent there in the Post-C test. ^#^*p* < 0.05 significant difference between STD-nonISD vs. STD-ISD. ^@@^*p* < 0.01 significant difference between STD-ISD vs. PF-ISD.

The ANOVA performed for the CPP data (see Figure 3A) showed a significant effect of the interaction between the variables Days × Housing × Stress (*F*_(2,85)_ = 3.198; *p* = 0.046; effect size 0.070). As expected, ISD animals housed in the standard condition (STD-ISD) developed CPP, since they spent more time in the drug-paired compartment in the Post-C test than in the Pre-C test (*p* < 0.01). This time was also significantly higher when compared to the time spent in the drug-paired compartment by animals housed with a female for long and short terms (PF, *p* < 0.05).

The ANOVA for the CS (see Figure 3B) also revealed an effect of the variable Housing × Stress (*F*_(2,85)_ = 3.235; *p* = 0.044; effect size 0.071). Once again, socially stressed mice under standard condition housing (STD-ISD) had higher CS when compared to non-stressed animals in the same housing conditions (STD-nonISD, *p* < 0.05) and when compared to stressed animals housed with a female for long and short terms (PF, *p* < 0.01).

### The Neuroinflammatory Response Induced by Intermittent Social Defeat Stress Is Reduced by Positive Social Housing Conditions

**Figure 4:**
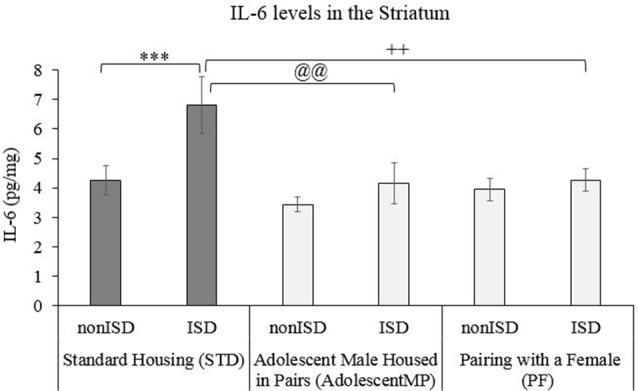
Housing condition modulates the increase in IL-6 levels induced by ISD after CPP. Biological samples were obtained from animals in the following groups: standard housing (STD-nonISD *n* = 12; STD-ISD *n* = 12); Male animals housed in pairs since adolescence (AdolescentMP-nonISD *n* = 8; AdolescentMP-ISD *n* = 8); Male animals paired with a female for long or short term (PF-nonISD = 16, PF-ISD = 16). The bars represent the mean values of IL-6 striatal levels (pg/mg) ± SEM. Bonferroni test ***p < 0.001 significant difference in IL-6 levels between STD-ISD vs. STD-nonISD. ^@@^*p* < 0.01 significant difference in IL-6 levels between STD-ISD vs. AdolescentMP-ISD. ^++^*p* < 0.01 significant difference in IL-6 levels between STD-ISD vs. PF-ISD.

A two-way ANOVA of IL-6 levels in the Striatum (see Figure 4) revealed an effect of the variable Housing (*F*_(2,66)_ = 4.490; *p* = 0.015; effect size 0.120). The *post hoc* test revealed that animals in STD housing condition had higher IL-6 levels than animals housed with other males since adolescence (AdolescentMP) or with a female (PF), *p* < 0.05 in both cases.

The ANOVA also revealed an effect of the variable Stress (*F*_(1,66)_ = 7.809; *p* = 0.007; effect size 0.106). ISD animals had higher striatal IL-6 levels when compared to the levels of non-ISD animals (*p* < 0.01).

Finally, the ANOVA also showed an effect of the interaction between Housing × Stress (*F*_(2,66)_ = 3.266; *p* = 0.044; effect size 0.090). Stressed animals under the standard housing condition (STD-ISD) had higher IL-6 striatal levels than non-stressed animals in the same housing condition (STD-nonISD; *p* < 0.001) than defeated animals housed in pairs since adolescence (AdolescentMP-ISD; *p* < 0.01) or those in the PF-IDS group (*p* < 0.01).

### Pairing With a Female (PF) Increases Plasmatic OT Levels

**Figure 5:**
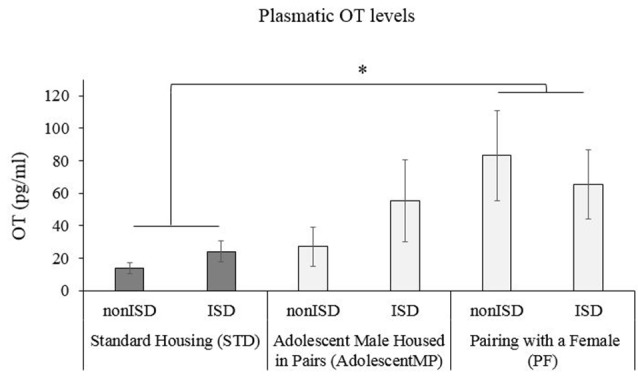
Short and Long-Term Pairing with a Female (PF) increases plasmatic OT levels. The bars represent the plasmatic OT levels of mice in Standard Housing condition (STD-nonISD *n* = 8; STD-ISD *n* = 8); male animals housed in pairs since adolescence (AdolescentMP-nonISD *n* = 8; AdolescentMP-ISD *n* = 8); Male animals paired with a female for long or short term (PF-nonISD = 16, PF-ISD = 16). The bars represent the mean values of concentration (pg/ml) ± SEM. *p < 0.05 significant difference in OT levels compared to Standard Housing (STD) condition.

A two-way ANOVA of plasmatic OT levels (see Figure 5) revealed an effect of the variable Housing (*F*_(2,58)_ = 3.154; *p* = 0.05; effect size 0.098). *Post hoc* test revealed a significant increase of plasmatic OT levels in animals housed with a female for long or short terms (PF) when compared to those housed in the standard condition (STD; *p* < 0.05).

## Discussion

The present research highlights how social factors are crucial in defining the individual’s response to cocaine. Negative social events, such as agonistic encounters between conspecifics, are powerful stressors capable of altering physiologic and psychological functions. Animals that undergo four sessions of an ISD protocol display long-lasting alterations, including an anxious phenotype, enhanced sensitivity to the rewarding properties of cocaine, and increased pro-inflammatory signaling in the striatum. On the other hand, we have also seen that social enrichment in the form of positive housing conditions has a protective effect by reducing the above-mentioned negative consequences of social stress. Animals housed in positive social conditions—for instance, in pairs with a female or with a familiar male—are buffered against the long-lasting increases in the rewarding properties of cocaine, anxiety-like behavior and the inflammatory response induced by ISD.

It is widely demonstrated that stressful social events have a modulatory effect on the effects of drugs (Gasparotto et al., 2005; Neisewander et al., 2012; Baracz et al., 2018). In our ISD protocol, the experimental mouse was confronted with a territorial (isolated) mouse that threatened and attacked the former, which adopted a defensive/submissive response (Miczek et al., 2008). Our results show that intruder animals experience these interactions as social stressors with a negative valence, and are subject to a series of long-lasting physiological and behavioral consequences that are consistent with previous evidence (Ferrer-Pérez et al., 2018a,b; Montagud-Romero et al., 2018). Socially defeated animals under standard housing condition (STD-ISD) showed increased sensitivity to the conditioned rewarding properties of cocaine, as they developed CPP for a subthreshold dose of cocaine (1 mg/kg), while the same dose was ineffective in animals that were not exposed to the ISD protocol (STD-nonISD). These animals also displayed an anxiety-like behavior phenotype characterized by spending less time in the open arms of the EPM than their non-stressed counterparts.

Rather than owing to a single mechanism, ISD effects are related with multiple and complex changes to peripheral and central systems that are not yet completely understood. Among these alterations, we hypothesize that immune response activation is critical in the appearance of the abovementioned long-lasting stress consequences. The results of the present study confirm this hypothesis by showing that socially defeated animals housed under standard condition (STD-ISD) display higher striatal levels of IL-6 than non-ISD animals. Following the same social defeat protocol, we have previously observed an increase in plasmatic and central levels of the pro-inflammatory cytokine IL-6 that returned to normal 3 weeks after the last defeat and increased again after cocaine-induced CPP (Ferrer-Pérez et al., 2018a). Therefore, we can affirm that intermittent social stress activates an initial immune response that promotes a sensitization of the neuroimmune axis and enhances the potential of cocaine to induce a pro-inflammatory state.

Previous studies indicate that positive social housing conditions are a successful intervention for reducing or preventing the negative consequences of social stress, mostly focus on anxiety behavior (Gasparotto et al., 2005; Nakayasu and Ishii, 2008; Neisewander et al., 2012). Housing with a female—thereby allowing mating behavior—for either long- (LTPF) or short-term (STPF) periods completely counteracted the anxiogenic effects of ISD and blocked cocaine-induced CPP, preventing the sensitization of the reward system by stress. Other researchers have reported that cohabitation with a female has a protective effect against the acquisition and extinction of cocaine CPP (Ribeiro Do Couto et al., 2009) and buffers against the anxiogenic effect of social and physical stress (Gobrogge and Wang, 2015). However, no studies have evaluated this cohabitation after exposure to social stress. We observed that this housing condition also exerted a protective effect against intermittent social stress-induced sensitization of the immune axis, as neuroinflammatory markers were not enhanced after the CPP procedure. We also observed that this housing condition induced a significant increase in plasmatic OT levels, which led us to suspect that this anti-inflammatory effect is mediated by the release of OT during social interaction in the cage. Although it is known that OT can exert an anti-inflammatory effect by decreasing the hypothalamic-adrenal-axis response to stressors (Lee et al., 2009; Karelina et al., 2011; Yuan et al., 2016), in our design, pairing with the female took place after the last defeat. Therefore, another mechanism that may explain the protective effect of this neuropeptide is that OT has the ability to change the focus from drug reward to social reward (McGregor and Bowen, 2012), thus enhancing the ability of a positive social stimulus to compete as an alternative reinforcer. In support of the role of OT in social defeat effects, we have recently reported that an injection of exogenous OT before each defeat episode induced a protective effect by blocking stress-increased anxiety-like behavior and the increased rewarding properties of cocaine in the CPP and the SA while favoring the extinction of drug memory (Ferrer-Pérez et al., 2019).

Free-living male mice prefer to live with females than with other males (Kappel et al., 2017). Group-housed male mice develop a social hierarchy, and under laboratory housing conditions it is common to witness inter-male aggression while dominance is established within the cage (Kappel et al., 2017). Indeed, aggressions can continue even after the establishment of a stable hierarchy, as a consequence of the alteration of territorial scent marking during the cleaning of cages (Poole and Morgan, 1973). Several research works have demonstrated that the status of an animal in the hierarchy of the cage modulates its vulnerability to the negative consequences of social stress. For instance, Yanovich et al. (2018) found that submissive animals are more likely to display anxiety-like behaviors and enhanced attraction to addictive substances when exposed to stress, while dominant animals are more resilient to the negative consequences of stress. Considering the negative effects of the continuous fight for dominance that characterizes standard housing, we designed a low hierarchic stress housing condition as a buffer against the consequences of social defeat stress. We paired two adult males (AdultMP) with the intention of promoting a more predictable hierarchy and thus reducing the stress derived from fights to establish dominance. Previously, other researchers have reported that the strategy of housing two familiar rats together prevents anxiety-like behavior induced by social stress (Nakayasu and Ishii, 2008). Conversely to previous reports and to our predictions, this pairing of adult mice failed to prevent the negative effect of ISD stress, as the animals in question displayed a similar CPP for a subthreshold dose of cocaine to that registered in defeated animals housed in standard condition (STD-ISD). We should take into consideration that Nakayasu and Ishii (2008) employed a single episode of social defeat, while our protocol consisted of intermittent defeat encounters over several days. It is possible that, given that these housing conditions were established upon arrival of the animals at the laboratory (42 PND), and that social defeat or exploration protocols began immediately after the acclimatization period (on PND 47), animals were experiencing social defeat stress while the cage hierarchy was still being established; in this way, both animals in each pair would have been experiencing the dominance/submission stress that is usually resolved after 21 days (Poole and Morgan, 1973; Rodríguez-Arias et al., 1998). We believe that this initial stress during the early definition of hierarchic positions was less evident in the standard housing condition because of the size of the group (four mice). Not all four animals in standard housing conditions directly experienced the stress of the dichotomy of submissive/dominant roles, as there were animals that occupied an intermediate hierarchic position. To test this hypothesis, we repeated the experiment, but this time established the housing conditions during adolescence, on PND 21 (AdolescentMP), so that social defeat and the exploration protocol would take place after the initial instability of hierarchy establishment. Now, this housing condition became a protective environment, in line with our earlier predictions. The stress-enhanced response to the rewarding properties of cocaine and sensitization to the neuroinflammatory response induced by ISD were both blunted. In this case, the mechanism underlying the protective effect of being housed with a familiar male since adolescence was not directly related to the OT buffering. We hypothesize that the protective effect observed in the AdolescentMP group was the result of a better resolution of the stress response, which limits the negative consequences of social defeat stress that are secondary to maintenance of the cage hierarchy.

The present research highlights how social interactions with conspecifics are powerful mediators of the individual’s response to drugs of abuse. All positive social housing conditions analyzed prevented the sensitization of the neuroimmune axis and the pro-inflammatory state induced by ISD. However, we were not able to prove the causal role of OT mediating this anti-inflammatory effect, as OT levels did not predict the variations observed in IL-6 concentration. Therefore, future research will be needed to identify the neurophysiological mechanism that underlies the buffering potential of positive social interactions against long-lasting ISD effects.

## Ethics Statement

The experimental protocol was approved by an Institutional Review Committee for the use of animal subjects (Comité d’ Ética d’Experimentació i Benestar Animal, number 2015/VSC/PEA/00168). Procedures involving mice and their care were conducted according to national, regional and local laws and regulations, which are in compliance with the Directive 2010/63/EU. All efforts were made to minimize the animals’ suffering and to reduce the number of animals used.

## Data Availability

The datasets generated for this study are available on request to the corresponding author.

## Author Contributions

CM, JM and MR-A designed, funded and administered the study. CM and JM reviewed the manuscript. CF-P, MR and MR-A designed, executed the study, analyzed the data, wrote and reviewed the manuscript.

## Conflict of Interest Statement

The authors declare that the research was conducted in the absence of any commercial or financial relationships that could be construed as a potential conflict of interest.
